# Vertical transmission of chikungunya virus: a worldwide concern

**DOI:** 10.1016/j.bjid.2024.103747

**Published:** 2024-05-06

**Authors:** Bárbara Silveira Faria, Lívia Barbosa da Silva, Clarissa Ferreira Rocha Avelar, Paula Antunes Souza de Morais, Aline Almeida Bentes

**Affiliations:** a Hospital Maternidade Sofia Feldman, Minas Gerais, MG, Brazil; b Hospital Infantil João Paulo II, FHEMIG, Minas Gerais, MG, Brazil

**Keywords:** Chikungunya fever, Arbovirus infections

## Abstract

The Chikungunya Virus (CHIKV) already has endemic circulation in about 100 countries and the number of infected patients increases every year, due to the effectiveness of the vector and human universal susceptibility to infection. The virus can also be transmitted from mother to child, more frequently intrapartum. About 50 % of neonates with CHIKV symptoms will have neurodevelopmental delay. It is therefore an infection of worldwide concern with a great impact on people's quality of life. The objective of this work is to describe two cases of confirmed vertical transmission by chikungunya virus, one of them with intrauterine infection and death of the neonate. Neonates with vertical chikungunya infection may present with clinical sepsis in the first few days of life, which is why this is a very important diagnosis, especially during outbreaks of the infection.

## Introduction

Chikungunya is an RNA virus of the *Alphaviru*s genus, transmitted to human and non-human primates by mosquitoes of the *Aedes* genus.^1^ Outbreaks have been described since the 1950s in Africa and Asia, with confirmation of vertical transmission in an outbreak that occurred on the island of Reunion in 2005.^1^^,^^2^ Since then, outbreaks have been described in several countries and currently there is endemic circulation in about 100 countries with more than 5 million confirmed cases in the last 15 years.^3^ In Brazil, in 2023, there were 143,739 probable cases with 82 deaths confirmed by the infection and an incidence rate of 67.4 cases per 100,000 inhabitants.^4^

The vertical transmission rate described in systematic reviews varies between 15.5 % and 50 % according to the gestational period in which the pregnant woman is infected.^5^^,^^6^ The neonatal disease is serious and resembles early sepsis with central nervous system involvement in about 60 % of infected infants.^5^^,^^6^ Studies that have evaluated the neurodevelopment of children exposed to CHIKV report a rate of developmental delay of 50 % in symptomatic infants.^7^ The infection also causes symptoms in 70 % of infected patients, which can lead to chronic arthritis, disabling between 15 %‒30 % of these, therefore it is one of the infectious diseases with the greatest potential for social and economic harm, and one of the most concern worldwide.^8^, ^9^, ^10^

Thus, the objective of this article is to describe two cases of vertical transmission confirmed by the Chikungunya Virus (CHIKV), one of which resulted in the death of the neonate, and to emphasize the importance of the differential diagnosis in cases of neonatal sepsis, especially in periods of outbreaks.

## Case reports

A 30-year-old pregnant woman, with no previous comorbidities, G2P1A0 (2nd pregnancy, 1 previous birth, 0 abortions), underwent routine prenatal care with ten visits. She developed Gestational Diabetes Mellitus (GDM), which was managed through diet. Prenatal serologies for HIV, syphilis, and hepatitis were non-reactive. She was susceptible to toxoplasmosis. Rapid tests for HIV, hepatitis B, and syphilis at the maternity ward were non-reactive.

In the last trimester, at a gestational age of 32 weeks and 2 days, she experienced fever, myalgia, and arthralgia in her hands. She sought medical attention, and a blood count revealed lymphopenia with 186 cells (3.5 %). She was discharged with instructions to use non-steroidal analgesics, without further investigations. On the 2nd day after symptom onset, her arthralgia worsened, accompanied by generalized body itching without identifiable rash. On the 5th day after symptom onset, she perceived reduced fetal movement, prompting another urgent medical visit.

Throughout the course of the illness, the pregnant woman remained hemodynamically stable. However, tests conducted at the maternity ward revealed altered cardiotocography with reduced variability and obstetric ultrasound showed absent fetal movement and tone, necessitating an emergency cesarean section due to the non-reassuring fetal state.

The cesarean delivery took place with ruptured membranes, clear amniotic fluid, a gestational age of 33 weeks and 3 days, birth weight of 2235 gs (p65), length of 45 cm (*z* = 0.33), and head circumference of 32 cm (*z* = 0.62). The newborn was born depressed, requiring resuscitation procedures and orotracheal intubation in the delivery room. Apgar scores at 1 min and 5 min were 4 and 4, respectively.

**Fig. 1:**
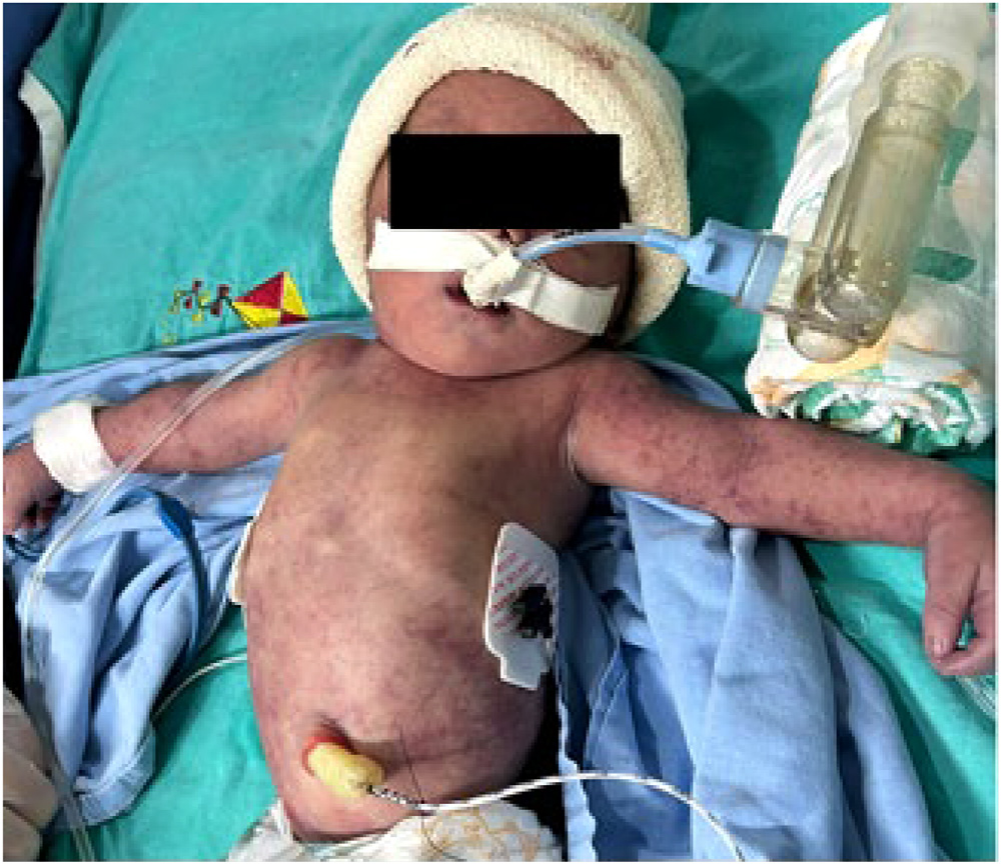
Petechial rash in a newborn with congenital chikungunya.

At birth, the newborn had petechial skin lesions (Fig. 1), focal seizures of the left upper limb, and hemodynamic instability necessitating vasoactive amines. Throughout the hospitalization, paired serologies were collected, and chikungunya serum Reverse Transcriptase Polymerase Chain Reaction (RT-PCR) for the mother-infant pair. The newborn required transfusions of fresh frozen plasma, cryoprecipitate, red blood cells, and platelets. The newborn developed massive intracranial hemorrhage and passed away at 34 h of life.

Laboratory tests on the newborn showed detectable serum chikungunya RT-PCR, non-reactive chikungunya IgM serology. The RT-PCR test was carried out at the State reference laboratory (FUNED-MG) using the 6856F/6981c/6919-FAM primer set for the nsp4 gene as the reference assay, designed by the Diagnostic and Reference Laboratory, Arbovirus Diseases Branch, CDC.^11^^,^^12^ CMV PCR in urine was undetectable. Maternal tests confirmed the congenital etiology, with chikungunya IgG serology non-reactive and chikungunya IgM serology reactive at 10.0 (reference value > 1.1). Serological tests for detection of IgG and IgM anti-CHIKV antibodies were carried out in a partner laboratory with a commercial immunoenzymatic test.^13^ Dengue IgM was non-reactive, Zika IgM was non-reactive, Parvovirus IgM was non-reactive, and Parvovirus IgG was non-reactive (Table 1).

**Table 1:** Laboratory test results.

**Exams**	**Newborn 1**	**Mother 1**	**Newborn 2**				**Mother 2**
	**At Birth**		**10th day of life**	**12th day of life**	**14th day of life**	**20th day of life**	
Hemoglobin (RV:14.5–22.5)	11.7		17.1	15.5	14.1	12.7	
Hematocrit (RV: 45‒67)	36.2		48.7	44.4	40.9	37.1	
Leucocytes (RV: 9.000 a 30.000)	7.018		31.140	23.070	19.890	19.360	
Mielocytes/Metamielocytes/Bastonocytes/Segmenteds	0/1/23/23		2/4/5/37	2/2/2/49	0/0/1/45	1/1/1/42	
Eosinophils/Limphocytes /Monocytes	0/45/7		0/32/20	1/36/6	0/40/10	1/39/15	
Platelets (RV: 200.000‒500.000)	56.000		43.000	24.000	87.000	431.000	
Hemoculture	NBG		NBG				
Prothrombin Time (RV: 9.3‒13.3)	52				13		
Prothrombin Activity (RV: 70 %‒100 %)	5.1 %				100 %		
Urea (RV: 11‒38)/Creatinine (RV: 0.2‒0.4)	25/0,9				21/0.5	34/1.1	
Transaminase ALT (RV: 15‒60)/AST (RV: 13‒45)	187/17				72/36	42/13	
Fibrinogen (RV: 125‒300)	INCG						
Lactate Dehydrogenase (LDH) (RV:180‒430)	1.522						
Albumin (RV: 3.5‒5.4)	0.7						
D-dimer (RV < 0.5)	4.59						
Liquor			Normal		Normal		
PCR Chikungunya Serum	DT						
PCR Chikungunya Liquor						UDT	
Chikungunya Serology							
IgG (Reactive: > 1.1)	IgM NR	IgG NR		IgG NR		IgG 1.8	IgG NR
IgM (Reactive: > 1.1)		IgM 10.0		IgM 8.7		IgM 8.5	IgM 10.3
Dengue Sorology		IgM NR					IgM IDT
Zika Sorology		IgM NR					
PCR CMV	UDT					UDT	
PCR herpes	UDT	‒					

CMV, Cytomegalovirus; DT, Detectable; IDT, Indetermined; IgG, Immunoglobulin G; IgM, Immunoglobulin M; INCG, Incoagulable; NBG, No Bacterial Growth; NR, Non-Reactive; PCR, Polymerase Chain Reaction; RV, Reference Value; UDT, Undetectable.

The second case involves a 44-year-old mother with no prior health issues, G3P2A0 (3 pregnancies, 2 previous births, 0 abortions), who underwent routine prenatal care with 8 visits. She had Gestational Diabetes Mellitus (GDM). Prenatal serologies for HIV, syphilis, and Hepatitis B were non-reactive. She was immune to toxoplasmosis. Rapid tests for HIV, HBsAg, and syphilis at the maternity ward were non-reactive. She had an uncomplicated vaginal delivery.

The postpartum woman experienced an isolated fever peak of 38.1 °C (immediate postpartum), with no other symptoms. A blood count was performed for infectious investigation, revealing lymphopenia (WBC: 6810 (Neut: 80 % | Lymph: 6 % | Mon: 14 %). She was discharged by the obstetric team within 48 h due to the absence of symptom recurrence. On the 5th day postpartum, she sought medical attention due to the appearance of diffuse petechiae on her body associated with itching, as well as pain in her lower limbs. A blood count showed leukopenia (WBC: 3870 (Neut: 66 % | Eos: 1 % | Lymph: 28 % | Mon: 5 %) (Table 1). Physical examination revealed a hardened lesion on her left leg, tender to palpation, and swelling of the limb. She was diagnosed with erysipelas and opted for hospitalization for observation and oral antibiotic therapy with amoxicillin-clavulanate. She was discharged from the hospital with partial improvement of symptoms.

She had a vaginal delivery, with rupture of membranes during labor, and clear amniotic fluid, without complications, at a gestational age of 39 weeks and 2 days. The baby's birth weight was 2975 gs (p21), with length of 51.5 cm (*z* = 1.2), and head circumference of 34.5 cm (*z* = 0.41). Apgar score at 1 min: 9 | Apgar score at 5 min: 10. The newborn remained hospitalized due to a sacral stigma, requiring neurological investigation with sacral ultrasound (no abnormalities found). On the 5th day of life, the newborn developed a fever (maximum temperature: 38.2 °C), refused feeding, exhibited hypoactivity, had weak pulses, and a diaper with streaks of blood. Infectious workup was initiated, and antibiotic therapy was started. On the 7th day of life, the newborn developed a maculopapular rash on the trunk and extremities and worsened hypoactivity. The newborn could not tolerate breastfeeding and had the last fever spike. Laboratory tests for infectious screening revealed lymphopenia, and blood cultures were negative. Antibiotics were discontinued considering the unlikely bacterial sepsis and a similar maternal condition. Given these circumstances, a viral investigation was pursued. A rapid dengue test was non-reactive, and paired serologies were requested.

On the 10th day of life, the newborn experienced recurrent fever spikes and irritability. A new infectious screening was initiated, showing thrombocytopenia and leukocytosis. The doctors prescribed antibiotics and performed a lumbar puncture on the baby, yielding unremarkable results. The patient showed clinical improvement, the rash disappeared, and there was better acceptance of the diet with successful breastfeeding. Antibiotic therapy was stopped, and the diagnosis of neonatal chikungunya was confirmed by immunoenzymatic IgM serology test at 8.7 (reactive) and non-reactive IgG13. Repeated chikungunya serology before hospital discharge to assess IgG turnover (Table 1).

The newborn had a prolonged hospital stay due to difficult-to-control painful crises, leading to irritability. Various medications were employed (NSAIDs, opioids, corticosteroids, and a GABA analog).

**Fig. 2:**
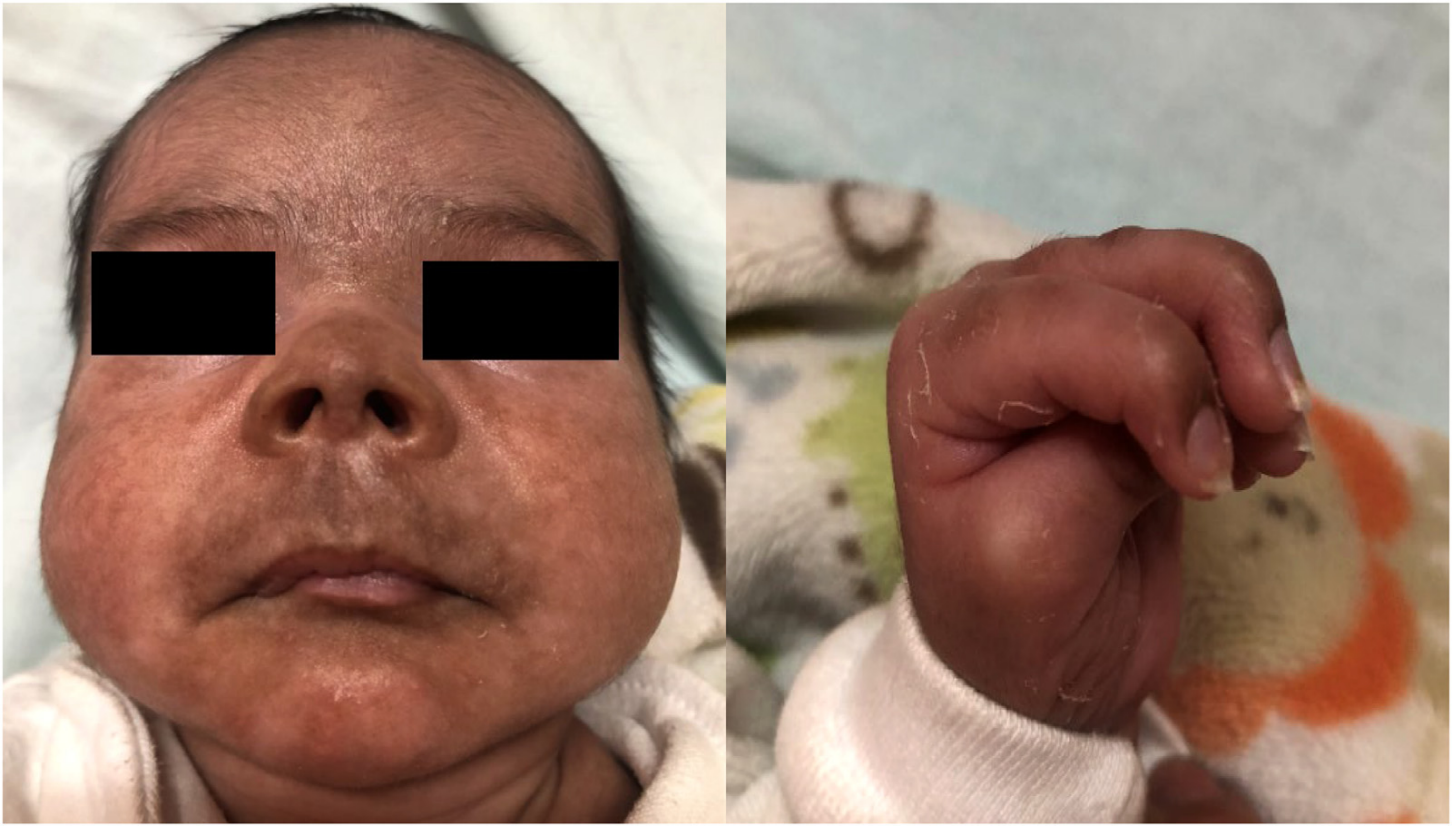
Hyperpigmented lesions on face and claw fingers.

The newborn displayed hyperpigmented facial lesions and edema in the feet and left-hand fingers in a claw-like configuration (Fig. 2). Additional tests were conducted on the newborn, including undetectable CMV PCR in urine, normal Ultrasonography of the Fontanelles (USTF), normal fundoscopy, and normal Brainstem Evoked Response Audiometry (BERA). A repeat chikungunya immunoenzymatic serology test was performed before discharge to assess IgG seroconversion, with a result of IgG 1.8 reactive (RV > 1.1) and IgM reactive 8.5 (RV > 1.1).^13^ Maternal tests revealed Chikungunya IgM at 10.3 (reactive), non-reactive IgG; reactive herpes IgG and non-reactive IgM; and reactive cytomegalovirus IgG and non-reactive IgM (Table 2).

**Table 2:** Main clinical manifestations and outcomes reported in studies of vertical transmission by CHIKV.

**Reference/Country**	**Number of neonates**	**Gender**	**Clinical manifestation**	**Treatment**	**Outcome**
Sahana M. Srinivas, G.C. Marlursiddappa Pradeep, 2018. India^14^	1	Male	11 days old fever, poor feeding, presumed sepsis, hyperpigmented macules on face and trunk, multiple small necrotic ulcers	Supportive measures and physiotherapy	Extensive dystrophic calcifications, atrophic scars and no deformities
Shen et al., 2021 China and Myanmar^15^	3	2 males and 1 female	Disseminated maculopapular rash, fever, drowsiness, arthralgia	Antibiotics, antypretic. The female baby received antiviral treatment (IFNB-1a 800,000 IU bid)	They progressed favorably and was discharged asymptomatic
Fajardo et al., 2021. Brazil^16^	2	Females	No symptoms in the first weeks of life. Developmental delay observed from 83 days of life.	Clinical monitoring of neurodevelopment	Neurocognitive delay up to 3 years of age
Sreekanth et al., 2022. India^17^	1	Female	Six-day old late onset sepsis, seizures, cranial hemorrhage, maculopapular rash, perioral hyperpigmenation	Phenobarbital, fresh frozen plasma, red blood cell transfusion, IVIG 1 g/Kg on the 7th and 9th days of admission	On follow up at three months, neurological examination was normal
Faustino et al., 2022. Brazil^18^	33	57.6 % male	Fever (79 %), myalgia (100 %), arthralgia (100 %) One presented neurodevelopment delay and one presented pos natal sepsis	Not described	At 3-months only one child presented neurological alterations
Torres et al., 2022. Brazil^19^	3	3 females	Fever, jaundice, thrombocytopenia, apnea, seizures, maculopapular rash	Antibiotics	One baby evolved with altered brain image on magnetic resonance
Sagay, et al. 2024. Nigeria^20^	26	Not described	Not described	Not described	Three stillbirths, two multiple congenital anomalies, one polydactyly with sepsis and jaundice, and one preterm

## Discussion

With the continuous rise in CHIKV cases in Brazil since 2019, it becomes crucial to increase clinical suspicion for this infection in pregnant women and investigate vertical transmission to infants. These clinical cases underscore the importance of investigating CHIKV infection in all pregnant women who present with fever during pregnancy, along with suggestive symptoms of arboviral infections such as maculopapular or petechial rash, arthralgia or arthritis, myalgia, or headache, associated or not with leukopenia in the blood count.^2^^,^^3^^,^^5^

Upon clinical suspicion, molecular biology tests (RT-PCR) for viral RNA detection should be collected from pregnant or postpartum women within eight days of symptom onset. Serological tests for the detection of IgM and IgG antibodies should be collected from the fifth day onwards and can be repeated 15 days later to assess the increase in IgG titers.^11^, ^12^, ^13^ Vertical transmission to the infant is confirmed through the detection of viral RNA (RT-PCR) collected from cord blood, serum, or cerebrospinal fluid, preferably within the first 24 h after birth, to rule out the remote possibility of infection through *Aedes* mosquito bites in the maternity ward.^2^^,^^5^^,^^6^ As infection often occurs during labor, the baby's serological tests should only be collected after one week of life, as symptoms of vertically acquired infection typically begin between three and seven days of life.^2^^,^^5^^,^^6^ In the cases described, the first case presented symptoms and detectable RT-PCR at birth, and the second case, while not having serum RT-PCR collected, showed reactive IgM and seroconversion of IgG. The mother in the second case had fever without a clear focus on the day of delivery and a blood count with lymphopenia, supporting the hypothesis that she was already infected on the day of delivery.

While there are well-documented cases of vertical transmission in the first and second trimesters of pregnancy, the majority of neonatal infections occur when the pregnant woman becomes infected within 15 days before delivery and four days after.^2^^,^^5^^,^^6^ Studies on Reunion Island have shown that vertical transmission of CHIKV can occur in about 50 % of cases when the pregnant woman has a high viral load during the early stages of labor.^2^ Abnormalities in fetal heart rate and meconium-stained amniotic fluid were common during labor in pregnant women with CHIKV viremia.^5^

Vertical transmission can occur intrauterine, intrapartum or peripartum, however, intrauterine infections by CHIKV are rarely described because the placenta is a protective barrier for fetuses during maternal viremia by this Alphavirus.^5^ However, we report here a case of a newborn with probable intrauterine transmission, who was born prematurely and very symptomatic, dying within 34 h of life and in which viral RNA was detected in the newborn's serum collected at birth. This baby's mother showed typical symptoms of CHIKV infection one week before premature birth. For reasons that are still unclear, in some cases the intense maternal viremia manages to overcome the placental barrier and infect the fetus while still in utero.

A systematic review that assessed 42 studies and 266 babies with confirmed vertical CHIKV infection found the following clinical manifestations: 94 % presented with a sepsis-like syndrome requiring ICU admission, 70 % had fever, 68.7 % exhibited neurological manifestations such as hypoactivity, irritability, meningoencephalitis, seizures, and intracranial hemorrhage; 55.2 % had dermatological lesions like maculopapular rash, hyperpigmentation, or bullous dermatosis; 51.5 % had cardiovascular manifestations, 46.2 % had hyperalgesia or diffuse limb edema, 41.7 % had respiratory symptoms, and 39.8 % developed motor, cognitive, or visual sequelae.^5^ Table two summarizes the studies not included in the systematic review on vertical transmission by Chikungunya in 2021^5^ (Table 2). There are studies on the topic published in America, Africa and Asia, but 25 % of them were published in Brazil, which represents the importance of this infection in the country.^4^^,^^16^^,^^18^^,^^19^

It's important to note that both infants reported here presented a similar picture of sepsis-like syndrome, rash, and neurological symptoms (focal seizures, intracranial hemorrhage, hypoactivity, and irritability). Biphasic fever was present in one of the cases, described as common in cases of chikungunya and characterized by no worsening of symptoms with defervescence, as described in dengue.^21^

The second baby had an exanthem followed by foot edema and facial hyperpigmentation. Hyperpigmentation and maculopapular rash are the most commonly documented dermatological manifestations. The hypermelanosis associated with chikungunya is a form of post-inflammatory hyperpigmentation, and increased intraepidermal melanin dispersion/retention triggered by the virus has been postulated as the cause of pigmentation. It presents as asymptomatic, dark brown macules, resembling freckles involving the central facial area, mimicking melasma-like lesions.^22^ The nose pigmentation was striking in several cases of CHIKV, which has not been reported in any other viral exanthem. Its presence and persistence for about three to six months after an infection assists in making a clinical and retrospective diagnosis of CHIKV infection.^23^

Only one study utilized a pain scale to monitor pain in infected newborns. Arthralgia was underreported in the evaluated articles due to the lack of pain assessment. Therefore, it is important to routinely establish neonatal pain monitoring in neonatal ICUs. Indeed, arthralgia should be evaluated in neonates, especially those with limb edema involving ankles and wrists. It was also proposed that feeding difficulties were due to painful jaw involvement, simulating trismus in some cases.^5^ The second case required prolonged hospitalization for pain management and the use of potent analgesics, as well as nasogastric tube feeding due to feeding difficulties.

This child, we did not observe clinical signs of arthritis, only swelling of the hands and feet that evolved with claw-like fingers in hands with normal neurological examination. Upon discharge, the child was referred to specialists to assess neuropsychomotor development and confirm possible joint sequelae. In the chronic phase of chikungunya, the most common symptom is persistent or recurrent joint involvement in the same joints affected during the acute phase, characterized by pain with or without edema, limitation of movement, deformity and absence of erythema. It is usually polyarticular and symmetrical.^24^

Other atypical manifestations may be present and are associated with a higher risk of death, such as hematological disorders and disseminated intravascular coagulation.^24^ The first described case presented the severe form of the disease with significant laboratory abnormalities and a fatal outcome.

The main laboratory finding of chikungunya reported in the literature is lymphopenia with a lymphocyte count below 1000 cells/mm^3^, as a finding related to viremia.^5^ Thrombocytopenia (platelets < 150,000 mm^3^) was a frequent laboratory finding in the cases reported here, associated with to the clinical picture of cerebral, gastrointestinal and conjunctival hemorrhages. Other laboratory abnormalities include elevated liver function tests and increased prothrombin time.^5^

Infection with the chikungunya virus is spreading rapidly because everyone who does not have antibodies is susceptible. Furthermore, among infants with congenital CHIKV infection, approximately 50 % of them evolved with developmental delay, in addition to an increased risk of death and prematurity. And there is still no specific treatment for this infection. Thus, there is great expectation for anti-CHIKV vaccines to be developed and approved for population use. There are several anti-CHIKV vaccine platforms being researched, but there is an attenuated vaccine that is in phase 3 of clinical trials. The vaccine, based on a live attenuated virus platform, induces the production of protective antibodies after a single dose, was well tolerated by the entire study population, and most adverse events were mild.^21^ However, while an effective vaccine is not released for population use, it is important to remember other forms of prevention that include environmental control of the vector and individual prevention with the recommendation to use repellents during pregnancy.

## Conclusion

Vertical transmission of chikungunya should be considered among the differential diagnoses when a newborn presents with clinical features suggestive of encephalopathy, blistering skin lesions, and perioral hyperpigmentation, especially in endemic areas for the disease. The clinical presentation resembling sepsis-like symptoms and laboratory abnormalities such as thrombocytopenia and lymphopenia do not allow differentiation from bacterial sepsis, and diagnosis becomes challenging when maternal infection has not been diagnosed.

Arboviral infections should be included in the questioning during the prenatal history-taking process with pregnant women and parturients. It is the role of the pediatrician to suspect the diagnosis, primarily based on the maternal history, and thus request virus testing for both the mother and the newborn. The discharge of the mother-baby dyad from the hospital should be carefully evaluated in cases where the mother exhibits signs of the disease before or immediately after delivery. Rigorous observation of perinatal disease signs and symptoms is essential, and even asymptomatic newborns should be kept hospitalized for a week to ensure better clinical and laboratory monitoring.

Efforts should focus on disease prevention, not only through vector control, but also by providing guidance to pregnant women during prenatal care regarding the use of repellents for individual protection.

## Conflicts of interest

The authors declare no conflicts of interest.
